# Cholinergic modulation of exploration in rats performing a spatial memory task

**DOI:** 10.1101/2023.10.14.559618

**Published:** 2023-10-14

**Authors:** Skylar Cassity, Irene Jungyeon Choi, Adeleke Malik Igbasanmi, Sarah Cristi Bickford, Kiara Tyanni Moore, Anna Elisabeth Seraiah, Lilly O’Shea, Dylan Layfield, Ehren Lee Newman

**Affiliations:** 1 *Department of Psychological and Brain Sciences*, College of Arts and Sciences, Indiana University Bloomington, 1101 E 10^th^ St., Bloomington, IN, 47405, USA; 2 *Intelligent Systems Engineering*, Luddy School of Informatics Computing and Engineering, University Bloomington, 1101 E 10^th^ St., Bloomington, IN, 47405, USA; 3 *Program in Neuroscience*, College of Arts and Sciences, Indiana University Bloomington, 1101 E 10^th^ St., Bloomington, IN, 47405, USA

**Keywords:** Acetylcholine, Medial septum, Rat, Navigation, Rearing

## Abstract

Spatial memory encoding depends in part on cholinergic modulation. How acetylcholine supports spatial memory encoding is not well understood. Studies of rearing onto hind legs, a representative exploratory behavior, suggest that acetylcholine release promotes rearing. Here, to test this hypothesis, we tracked rearing while optogenetically modulating the activity of choline acetyltransferase containing (i.e., acetylcholine producing) neurons of the medial septum in rats performing a spatial working memory task (n=9 rats). The cholinergic neurons were optogenetically inhibited using halorhodopsin for the duration that rats occupied two of the four open arms during the study phase of an 8-arm radial arm maze win-shift task. Comparing rats’ behavior in the two arm types showed that rearing frequency was not changed but the average duration of rearing epochs became significantly longer, and the total distance traveled increased in arms wherein the cholinergic neurons were inhibited. This effect on rearing and locomotion was only observed when the optogenetic inhibition was performed during the study phase. Inhibiting the cholinergic neurons during the holding phase prior to trial onset had no significant effect on any metrics tested. These results indicate that cholinergic neurons in the medial septum have a context dependent effect on exploration, do not promote rearing directly, but may facilitate the rapid encoding of information while rearing.

## 1| Introduction

Determining the mechanisms that support the encoding of new memories remains a major outstanding challenge. Spatial memory is a focus of investigations in the neurobiology of memory in the context of rodent models of memory. Acetylcholine is thought to be important for encoding spatial memories (Whishaw et al., 1985, Blokland et al., 1992; Elvander et al., 2004; Martyn et al., 2012; but also see Parent & Baxter, 2004). Yet, the mechanisms by which acetylcholine supports memory encoding remain unclear.

A dominant hypothesis for how acetylcholine supports memory encoding is that it enacts a switch of functional modes, promoting processing of bottom-up, sensory, information (for relevant reviews, see Hasselmo, 2006; Newman et al., 2012; Honey, Newman, Schapiro, 2017). This hypothesis is supported by circuit physiology studies indicating that cholinergic modulation increases the relative strength synapses from bottom-up projections relative to lateral projections (e.g., Hasselmo & Schnell, 1994; Giocomo et al., 2005; Newman et al., 2013; Douchamps et al., 2013; Dannenberg et al., 2015). This switch in the circuit dynamics allows high fidelity encoding of information carried into the circuit by extrinsic fibers by limiting interference from intrinsic projections. What is not addressed by this hypothesis is whether acetylcholine, beyond modulating the circuit dynamics, is also responsible for aligned exploratory behaviors.

The role of acetylcholine for modulating exploratory behaviors is not well understood. However, existing data suggest the working hypothesis that cholinergic tone promotes exploratory behavior. Supporting evidence comes from the study of genetic variations corresponding to increased exploratory behavior in mice (van Abeelen, 1989) and the study of cholinergic tone during the initial exploration of novel enclosures (Acquas et al., 1996; Thiel et al., 1998; Giovannini et al., 2001). In both, there is a positive relationship between exploration and cholinergic signaling. Yet, these correlational studies do not address whether acetylcholine drives the exploration or is coincident with it. Stronger evidence for causation comes from the study of exploratory behavior following local infusions of the cholinergic agonist carbachol into the septum (Monmaur et al., 1997; cf. Elvander et al., 2004). Monmaur et al. (1997) observed that, following agonist infusion, rats reared onto their hind legs more frequently when left in a narrow open cylinder without an overt task. Elvander et al. (2004) did not replicate this effect. However, they used a larger testing enclosure and instead found a significant increase in the total distance traveled which may nonetheless reflect increased exploration. Critically, however, neither examined exploration in the context of a memory paradigm.

Exploratory behaviors including rearing can be motivated by different objectives. For example, an animal may rear to plot an escape or to get oriented (Lever et al., 2006). Accordingly, exploration may be driven by different systems under different motivations. Thus, given the working hypothesis that acetylcholine supports spatial memory through the modulation of exploration for encoding purposes, it is important to specifically test the relationship between cholinergic modulation and exploration in the context of a spatial memory task. Rearing during the study phase of the 8-arm radial maze win-shift task is a relevant key epoch of spatial memory encoding (Layfield et al., 2022) making this a task well suited to the goals of the present study.

In the present study we test the hypothesis that acetylcholine is a physiological mechanism that drives rearing in the 8-arm radial maze win-shift task. This was accomplished by testing the effect of optogenetically inhibiting medial septal cholinergic neurons on rearing behavior and total distance traveled in rats performing an 8-arm radial maze task. Cholinergic neurons of the medial septum (MS) were targeted as these are the major projection of acetylcholine to the hippocampus (Leranth & Frotscher, 1985), which is of relevance because the hippocampus is the putative site of spatial memory encoding in the 8-arm win-shift paradigm (Olton & Samuelson, 1976; but also see Layfield et al., 2020). The use of optogenetic techniques permitted within trial manipulations, allowing us to compare rearing in arms when septal cholinergic neurons were unmanipulated rearing in arms when the neurons were optogenetically inactivated. Our logic is as follows: if the activity of these cholinergic neurons promotes rearing, then acute inhibition of their activity Counter to our working hypothesis, we show that reducing the activity of the cholinergic neurons increased both rearing duration and total distance travelled and had no significant impact on the frequency of rearing.

## 2 | Methods

All procedures and surgeries were conducted in strict accordance with National Institutes of Health and Indiana University Institutional Animal Care and Use Committee (IU IACUC) guidelines.

### 2.1 | Subjects

Ten *Chat*:: *Cre*+ Long Evans rats (Witten et al., 2011) were used for this study (6 male, 4 female). One female was removed from the study for failing to reach behavioral criterion. All were maintained on a 12 hours light / dark cycle. Nine rats were used for Experiment 1 of the study and six of the original nine were used for Experiment 2. Rats were bred in lab and identified as CHAT::Cre+ by ear punch genotyping (TransnetYX). Rats were individually housed with ad libitum access to water and food restricted to ~90% of free feeding weight.

### 2.2 | Behavioral Training

Behavioral training took place in a custom 8-arm radial maze with computer controlled pneumatic drop doors at the entrances. The maze had a 33.2 cm wide hub and each arm measured 48.26 cm long, 10.79 cm wide with 20.95 cm tall walls. Walls were made from clear acrylic to allow for visual orientation to distal cues that surrounded the maze. The floors were made from opaque matte white acrylic. The maze was open topped to allow testing of rats tethered by the fiber optic patch cable. At the end of each arm were food wells in which 45 mg sucrose pellets (Bio-Serv, Flemington, NJ) were delivered. The maze was surrounded by rich visual cues to facilitate allocentric orientation. This included objects taped onto the walls, furniture (bookshelf and desk), and the holding pedestal on which rats were placed between trial phases immediately adjacent to the maze. The experimenter was present in the room during all sessions, standing at the same location and wore the same PPE (white lab coat) during all sessions to maintain a stable distal cue.

#### 2.2.1 | Habituation & Preliminary Training

Rats were handled for ten minutes and given 20–30 sucrose pellets to habituate them to experimenter handing and rewards for 3 days prior to preliminary training. Preliminary training lasted for three days, with one session per day to introduce collection of sugar pellet rewards from the 8-arm maze. Three sugar pellets were placed along all eight maze arms leading from the center hub to feed dishes baited with two sugar pellets. Rats were placed in the center hub of the maze with all doors open and allowed to forage for ten minutes or until all sugar pellets were consumed. After each session, maze floors, walls, and doors were cleaned with 10% chlorohexidine solution to ensure cleanliness of the maze and reduce odors which might provide smell cues.

#### 2.2.2 | Initial training

Rats underwent initial training for ten days, one session/day and one trial/session. During each, rats were trained that all arms were baited with two sugar pellets each day. Training began when the rat was placed in the center hub and every door was promptly opened. Rats were allowed to forage until all pellets are collected or 15 minutes has elapsed, whichever came first. The time the rat was kept in the center hub before each door opened was increased incrementally in following sessions by ten seconds until one minute was reached.

#### 2.2.3| Task Training

##### Task:

**Figure 1:**
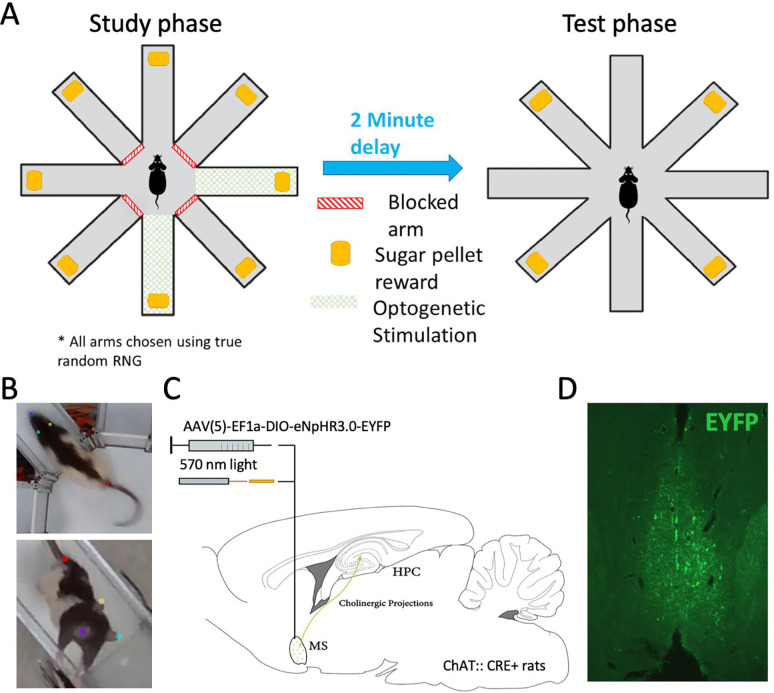
Overview of experimental approach. **A.** Rats performed a win-shift task on the 8-arm radial arm maze. A new random set of four arms opened in the study phase of each trial. Rats collected sugar pellet rewards from each. Two of the arms were randomly assigned to be the stim arms such, when the rat entered those arms, the experimenter activated the laser. In a two-minute delay following the study phase, rats were removed from the maze as it was cleaned. In the test phase all eight arms were opened and rats could collect reward from the unvisited four arms. **B.** Rat position was tracked with DeepLabCut to measure total locomotion. Rearing behavior (shown on bottom) was manually scored. **C.** The CRE dependent AAV5-EF1a-DIO-eNpHR3.0-EYFP virus was infused and a glass fiber was implanted into the medial septum of ChAT:: CRE+ rats to enable optogenetic inhibition of acetylcholine producing neurons. **D.** Representative histological section from a rat from this study showing EYFP labeling of neurons in the medial septum.

The spatial memory task used here was the delayed-win-shift task on an 8-arm radial arm maze. The task consisted of three phases, a study phase, a delay phase, and a test phase as shown in Fig. 1A. Prior to the study phase, the rat was placed in the central hub with all doors shut for 60 seconds. After the 60 seconds, a random set of four doors opened and the rat was allowed to collect pellets from each. The rat was then removed from the maze and placed on pedestal next to the maze for a two-minute delay phase. During the delay, the maze was cleaned with chlorhexidine. The test phase began by placing the rat in the hub and. After 60 seconds, all eight doors opened. The four arms that had not opened during the study phase baited food wells. The test phase ended after all the pellets had been consumed or when 15 min elapsed. Regular training (~5 days/week) continued until rats achieved behavioral the criterion of no more than three errors over four days.

### 2.3| Behavioral scoring

The primary dependent measures of spatial memory performance were percent correct and number of arm entries. Percent correct was measured as the number of the first four arm entries in the test phase that contained rewards divided by four. Number of arm entries was measured as the total number of arms entered to find all four baited reward sites in the test phase. A visit to an arm was defined to occur when the rat’s hind feet entered the arm. An arm visit counted as an error if the arm had been visited earlier in the trial. Secondary analyses also examined ‘reentries.’ Reentries were quantified as the number of times in the test phase that a rat entered an arm that had been open in the study phase arm.

### 2.4| Exploration scoring

The ray behavior was tracked by applying DeepLabCut (Mathis et al., 2019; Nath et al., 2019) to video recordings of the rat in the maze collected from an overhead camera (RGB sensor of a RealSense Depth Camera D435; Intel). The camera captured the whole of the maze at 30 Hz and 640 × 480 resolution. A DeepLabCut network was built with 24 frames from each of the 10 rats (240 total frames) labelled to mark the nose, left ear, right ear, and tail base (rump). Examples are shown in Fig. 1B. After an initial ~300k training iterations, an additional 240 ‘jumpy’ frames were extracted, relabeled, and added to the training set. The network then received another ~600k training iterations on the expanded training set. Network performance was then confirmed by manual inspection by checking labelled videos and quantifying jumpy frames in the extracted tracking data. Prior to use, the tracking data was preprocessed to interpolate over jumps of >5 cm from one frame to the next and to apply a Kalman Filter as implemented in the CMBHOME toolbox (https://github.com/hasselmonians/CMBHOME). Rat position (e.g., summing up total movement) was estimated by the position of the rump. Rearing events were manually scored through offline analysis by experimenters. Rearing events began once at least one forepaw left the maze floor and ended once both forepaws returned to the ground.

### 2.5| Surgery

After meeting behavioral criterion, rats underwent stereotaxic surgery to allow optogenetic inactivation of medial septal cholinergic neurons. Rats were anesthetized with 1–4% isoflurane in oxygen, scalp shaved and placed into a stereotaxic frame (Kopf Instruments). A scalp incision from anterior to posterior was made across the skull. A craniotomy for the viral injection and optical fiber was placed at [AP +06. mm, ML +1.0 mm]. A microinjection robotic stereotaxic arm (Cambridge Neurotech) at a 9° angle to avoid midline vascular structures was used to perform a microinjection of 2.5 μl halorhodopsin (AAV(5)-EF1a-DIO-eNpHR3.0-EYFP) (UNC Vector Core) to the medial septum [AP +0.6mm, ML +1.0mm, DV 6.1]. An optical cannula (NA=0.66) was lowered into the same area as the needle, delivered to rest 300–500 microns above the viral injection and fixed with bone screws inserted into the skull with dental acrylic. The site was suture closed around the implant. Rats recovered for 7 days before moving on to the next phase. A summary is shown in Fig. 1C.

### 2.6| Optogenetic control

Optogenetic control was implemented with the eNpHR3.0 halorhodopsin which inhibits neural activity with photostimulation (Gradinaru et al., 2010). Activation of halorhodopsin was achieved using light from a CE:YAG laser diode optical head laser system (Doric) filtered to 570 nm to 615 nm. Laser output was delivered to the medial septum by way of a patch fiber connected to a rotary joint (Doric) and, from the rotary joint a dual fiber optic patch cord (Doric) that was coupled to the implanted optical fibers prior to each testing session. Light intensity was controlled by the Doric Neuroscience studio software to obtain 5–10 mW at the tip of the fiber in the brain using a photodiode power sensor coupled to a power meter (Thorlabs). Laser activation was triggered by the experimenter via an external control signal generated by an Arduino unit (Arduino due).

Individual trials were assigned to one of two conditions: Stim and Control. Control trials were defined as trials with no light delivery. Stim trials varied between the two experiments. In Experiment 1, Stim trails consisted of optogenetic inhibition while rats were in either of two out of the four available arms in the study phase. New arms were chosen on each trial in a random fashion (Random.org True Random Number Service). No light was delivered on the other two arms. In Experiment 2, Stim trials consisted of optogenetic inhibition throughout the hub retention phase wherein the rats waited in the maze hub before the random four doors opened for the study phase. In no condition was optogenetic inhibition activated in any phase other than the study phase. Optogenetic input was triggered by the experimenter when the rat’s head broke the beginning plane of the arm, defined by the small gap between the floor of the center hub and floor of the arm. Rats were connected to the optical patch cords throughout all phases of testing regardless of trial type. Trial type was randomized from one day to the next in both experiments. Each rat completed 10 trials of each type in Experiment 1 before completing 8–10 trials of each type in Experiment 2.

### 2.7 | Histology

Upon completion of testing, animals were euthanized via isoflurane overdose and perfused intracardially with phosphate buffered saline (PBS) followed by a 4% paraformaldehyde saline solution. Brains were saturated with a 30% sucrose solution prior to sectioning. Coronal sections (40 um thick) were cut with a microtome (American Optic company). Immunohistochemistry was performed on free-floating sections to amplify induced EYFP reporter signaling. Sections were first rinsed with PBS then blocked with buffer (PBS, 5% normal goat serum, and 0.4% Trition X-100). This was followed by overnight incubation with conjugated anti-GFP rabbit antibody (1:2000; catalog no. A21311; Invitrogen). Finally, the sections were rinsed with PBS, mounted on slides, and cover slipped with DAPI and Fluoroshield. Labelled sections were imaged with an epifluorescent microscope. An example section is shown in Fig. 1D.

### 2.8| Statistical Analysis

Hypothesis testing was performed using single sample two-tailed t-tests. Significance was defined at the α= 0.05 level. Reported values indicate mean +/− standard error.

## 3| Results

Our goal was to test whether rearing changed significantly following halorhodopsin mediated inhibition of cholinergic neurons in the medial septum as rats completed the study phase of an 8-arm radial arm maze win-shift task. We explored this in two Experiments with the same rats.

In Experiment 1, we analyzed the frequency of rearing, duration of rearing, and distance traveled in arms in which light was delivered relative to arms in which no light was delivered. We also compared observed differences to those observed on control trials wherein the laser was not powered on. Our primary working hypothesis, based on prior reports, was that these cholinergic neurons directly control exploratory behavior and, accordingly, that inhibiting cholinergic neuron activity would reduce exploratory behavior.

**Figure 2:**
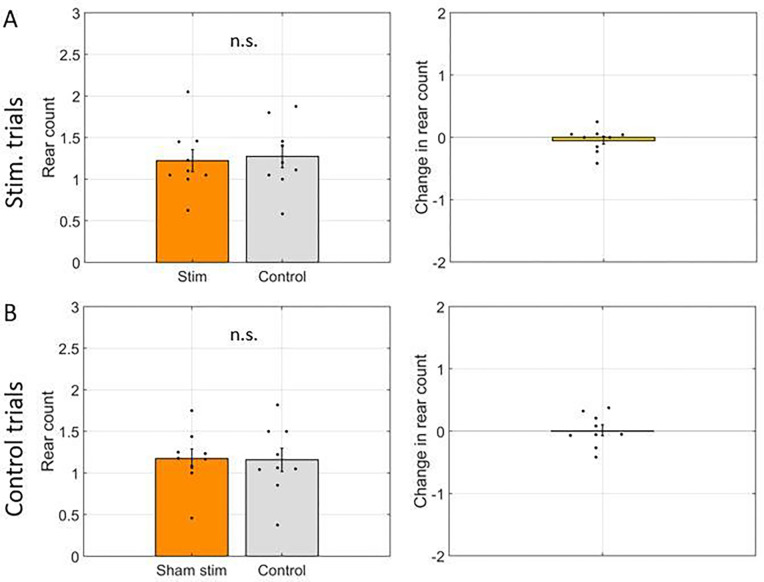
Rear frequency was not changed by optogenetic inhibition. **A.** Comparison of average number of rears per arm (rear count) between arms with laser activation (Stim) and arms without laser activation (Control). Left: Rear frequency in each arm type. Right: Difference in rear frequency between arm types. **B.** Same as A but for control trials wherein the laser was not powered on (Sham stim) and thus no inhibition was performed. In all panels, dots indicate the average over trials for individual rats, bar height indicates the mean over rats, and error bars indicate standard error over rats. n.s. = not significant.

We first asked if rearing frequency, quantified as the mean number of rearing events in each arm, differed significantly between arms when the laser was activated (‘stim arms’) and not activated (‘control arms’). The mean+/−std. err. was 1.22 +/− 0.14 rears per stim arm and 1.27 +/− 0.13 rears per control arm. The difference of −0.04 +/− 0.06 rears was not significantly different from zero (t(8) = −0.79, p = 0.45). The data for this comparison are shown in Fig. 2.

**Figure 3:**
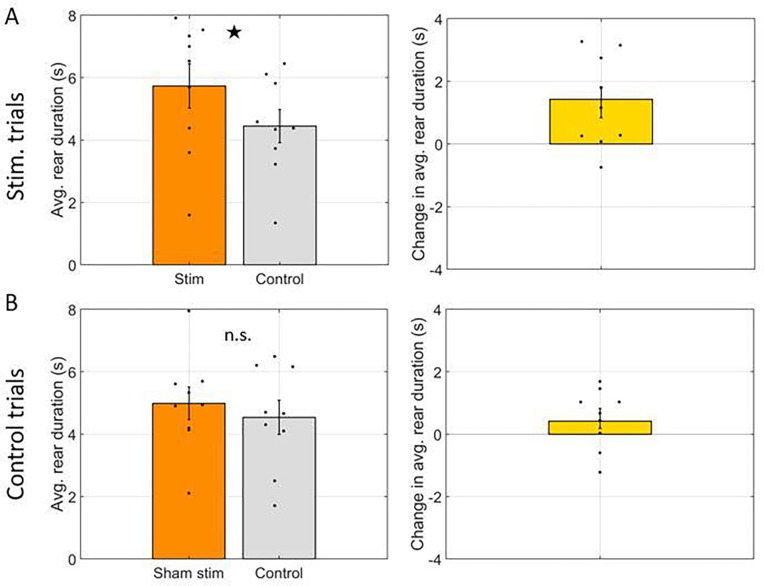
Rear duration was increased by optogenetic inhibition. **A.** Comparison of average duration of each rear between arms with laser activation (Stim) and arms without laser activation (Control). Left: Rear duration in each arm type. Right: Difference in rear duration between arm types. **B.** Same as A but for control trials wherein the laser was not powered on (Sham stim) and thus no inhibition was performed. In all panels, dots indicate the average over trials for individual rats, bar height indicates the mean over rats, and error bars indicate standard error over rats. ★ indicates p < 0.05 and n.s. indicates not significant.

We next asked if rear duration, quantified as the mean duration of rearing events, differed between stim arms and control arms. Rear duration was 5.67 +/− 0.71 seconds per rearing event in stim arms and 4.41 +/− 0.54 seconds per rearing event in control arms. The mean increase of 1.32 +/− 0.47 seconds per rear was significantly greater than zero (t(8) = 2.79, p = 0.02). This effect was specific to trials wherein the laser was powered on. Repeating the same analysis for control trials, wherein the laser was not powered on and thus did not deliver light in ‘stim arms’ revealed no significant change (difference of 0.41 seconds, t(8) = 1.49, p = 0.17). The data for this comparison are shown in Fig. 3.

**Figure 4:**
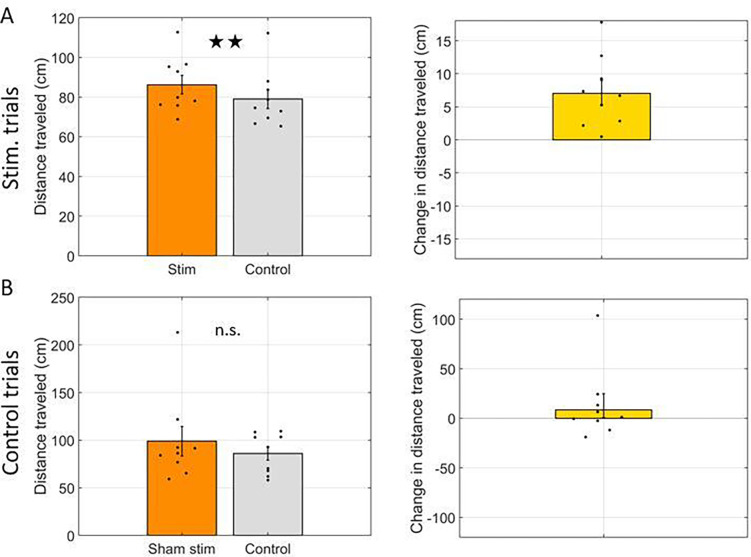
Total distance traveled was increased by optogenetic inhibition. **A.** Comparison of total distance traveled in arms with laser activation (Stim) and arms without laser activation (Control). Left: Total distance traveled in each arm type. Right: Difference in total distance traveled between arm types. **B.** Same as A but for control trials wherein the laser was not powered on (Sham stim) and thus no inhibition was performed. In all panels, dots indicate the average over trials for individual rats, bar height indicates the mean over rats, and error bars indicate standard error over rats. ★★ indicates p < 0.01 and n.s. indicates not significant.

Finally, we asked if distance traveled, quantified as the summed path length while in the arm, differed between stim arms and control arms. Distance traveled was 85.9 +/− 4.6 cm in stim arms and 78.7 +/− 4.5 cm in control arms. The mean increase of 7.2 +/− 1.6 cm was significantly greater than zero (t(8) = 4.52, p < 0.01). No such difference was observed in the control trials (97.6 +/− 14.7 cm in stim arms vs. 86.15 cm in control arms; t(8) = 1.00, p = 0.35). The data for this comparison are shown in Fig. 4.

Given acetylcholine’s role in encoding of spatial memories and the assumed role of rearing in spatial orientation, we also tested if modulating cholinergic neuron activity impacted memory performance. To do so, we compared memory performance in the test phase between trials where some stimulation was delivered during the preceding study phase (stim) and trials where stimulation was not delivered during the preceding study phase (control). Percent accuracy (scored as percentage of first four arm entries were rewarded) was not significantly changed (85.35% +/− 3.14% on stim trials vs. 84.02% +/− 2.07% on control trials; t(8) = −1.01, p = 0.34). Number of arm entries to collect all four rewards also did not change significantly (4.96 +/− 0.31 arm entries for stim trials vs. 5.38 +/− 0.33 arm entries for control trials; t(8) = 1.56, p = 0.16). We also examined stim trials to test if rats were more likely to re-enter stim arms at test than control arms. We found no significant difference (average number of re-entries was 0.54 +/− 0.09 for stim arms and 0.59 +/− 0.18 for control arms; t(8) = 0.34, p = 0.74).

In Experiment 2, we again analyzed the frequency of rearing, duration of rearing, and distance traveled but this time focusing on the rat behavior while waiting for 60 seconds in the hub prior to the start of the study phase. Our working hypothesis was that modulation of the cholinergic neurons would have different effects on exploration when the rat was in a different behavioral. Our rationale was that exploration done in the hub prior to trial onset is done in the absence of any trial specific information given that the rat has set to have the chance to see which arms will be opened in the present study phase. This is distinct from exploration we studied in Experiment 1 which was done after learning which arms were available during the study phase. For this experiment, the comparison was between trials: stim trials—wherein the laser was activated throughout 60 seconds prior to the doors opening—and control trials—wherein the laser was not activated.

Again, we found no difference in rearing frequency (4.1 +/− 1.15 rears in stim trials vs. 3.9 +/− 1.12 rears in control trials; t(6) = 0.10, p = 0.93). This time we found no change in rear duration (3.27 +/− 0.52 seconds in stim trials vs. 3.97 +/− 0.64 seconds in control trials; t(6) = −0.85, p = 0.42). We also found no change in the distance traveled (331 +/− 22 cm in stim trials vs. 318 +/− 21 cm in control trials; t = 0.42, p = 0.69). These results indicate that there was no effect of inhibiting the cholinergic neurons of the medial septum on exploratory behaviors during the pre-trial phase.

## 4| Discussion

This project aimed to test if the activity of cholinergic cells of the medial septum promoted rearing behavior in the context of a spatial working memory task. This hypothesis was motivated by previous correlational studies of exploration and cholinergic tone (Flicker & Geyer, 1982; van Abeelen, 1989; Acquas et al., 1996; Thiel et al., 1998; Giovannini et al., 2001; see Lever et al., 2005 for a related review). Such a connection, if it existed, could provide an explanation for the association between cholinergic modulation and spatial memory encoding given that rearing itself is a key epoch of spatial memory encoding (Layfield et al., 2022). Arguing against this connection however, we found that optogenetically inhibiting the activity of cholinergic neurons in the medial septum did not change rearing frequency. Rather, the optogenetic inhibition significantly increased rearing duration and total distance traveled.

The pattern of empirical findings reported here, a combination of no change in rearing frequency and an increase in rearing duration and movement in stimulation arms, argue against a simplistic hypothesis that activity of cholinergic neurons in the medial septum motivate rearing behavior. Such a hypothesis would have predicted reductions in both exploration and rearing.

Nonetheless, these results should not be interpreted as indicating that there is no relationship between acetylcholine and exploration. Indeed, given the rich literature on the importance of acetylcholine for encoding (for relevant reviews, see Hasselmo, 2006; Newman et al., 2012; Honey, Newman, Schapiro, 2017), it is reasonable to hypothesis that the importance of acetylcholine is down-stream rather than up-stream of the exploration. That is, acetylcholine does not motivate information collection but rather supports the effective encoding of the information once collected. By this view, the observed increase in rear duration during optogenetic silencing of the cholinergic neurons reflects the increased time needed to encode the necessary information. By analogy, this is like an increased reaction time to a more demanding stimulus. It takes longer to reach the same end state. The fact that we observed no significant change in behavioral performance in stim trials is consistent with the idea that the rats indeed reached the same end state.

The optogenetic inhibition used here should be regarded as a decrease in cholinergic tone, not a silencing of cholinergic tone. This is because the *ChAT:: CRE+* rats used to enable expression of halorhodopsin selectively in acetylcholine producing neurons have been found to only express CRE in ~50% of the ChAT+ neurons of the medial septum (Witten et al., 2011). While complete silencing would also have been fine, the objective of this study was achievable using the graded decrease.

The current pattern of results is at odds with at least one previous study of rearing in the context of cholinergic modulation but is methodologically distinct in several important ways. Monmaur et al. (1997) observed rearing frequency increased following a local infusion of the cholinergic agonist carbachol into the septum and observed increased rearing in rats being held in a narrow chamber. The infusion of carbachol into the septum would be expected to modulate all local receptors including sensitive mainly to cholinergic input from the brain stem. Here, by modulating the activity of cholinergic neurons located in the medial septum we would have had an impact at only a subset of all local receptors in addition to changing cholinergic tone in the hippocampus and entorhinal cortex. The work by Monmaur et al. also did not have the animals situated in a task and thus it is unclear what behavioral motivation the rearing may have been related to. For example, the rats may have been focused on looking for opportunities to escape. Here, we were specifically interested in spatial memory encoding.

Supporting the idea that differences in behavioral context could impact the relationship between cholinergic tone and rearing, we found here that optogenetic inhibition only increased rear duration and distance traveled in when rats were underway in the study phase. When we repeated the optogenetic inhibition manipulation while the rats waited for the study phase to start in the maze hub, we found change in neither rear duration nor distance travelled. By this line of thinking, rearing in the hub prior to the start of the study phase may be motivated by a different behavioral motivation that rearing that occurs in the arms once the study phase has started. For example, prior to the start of the trial, the rat knows that a subset of the doors out of the hub will open but not which. Once the trial has started and the rat is in an arm, the uncertainty of which arms will open is gone and now the rat is in a specific arm. Thus, cholinergic modulation may play a different functional role in these settings (e.g., Yu & Dayan, 2005).

In summary, the present work failed to find support for the hypothesis that activity of acetylcholine producing neurons in the medial septum motivate rearing. Rather, the findings show that inhibiting these neurons has no effect on rearing frequency but increases rearing duration. The increase in duration could be explained by the rats requiring more time to perform the same encoding. Finally, the current results support the hypothesis that rearing is multiply determined as the same optogenetic inhibition manipulation had different effects on rearing in different behavioral contexts.

## Figures and Tables

**Figure 5:**
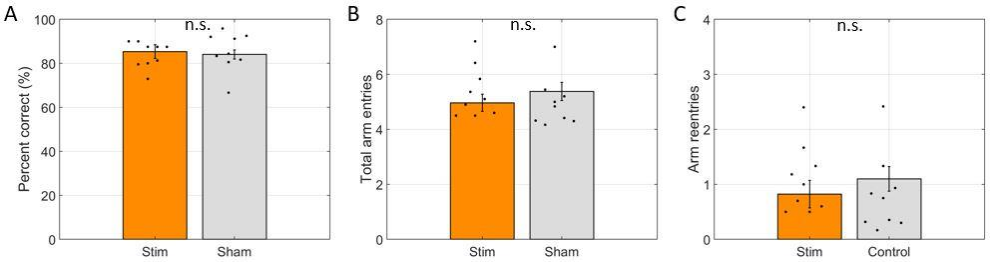
Win-shift performance was not changed by optogenetic inhibition. **A.** Percentage of the first four arms entered containing reward in the test phase (Percent correct) for trials with the laser powered on during the study phase (Stim) and trials with the laser powered off (Sham). **B.** Number of arms entered to find all four reward in Stim and Sham trials. **C.** Error rate (Arm reentries) for stim trials into stim arms and control arms. In all panels, dots indicate the average over trials for individual rats, bar height indicates the mean over rats, and error bars indicate standard error over rats. n.s. indicates not significant.

**Figure 6:**
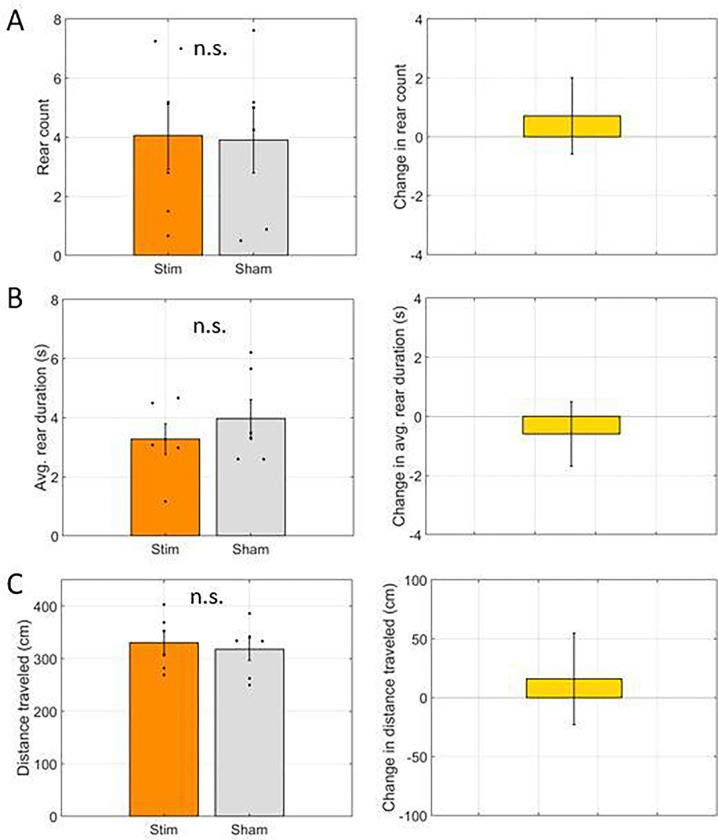
Exploration in the hub during the 60 seconds prior to start of the study phase was not changed by optogenetic inhibition. **A.** The number of rears in trials where the laser was powered on (Stim) were not significantly different than the number in trials where the laser was not powered on (Sham). **B.** The average duration of individuals rearing events occurring in the hub was not significantly different between stim and sham trials. **C.** The total distance traveled while waiting in the hub was not significantly different between stim and sham trials. In all panels, dots indicate the average over trials for individual rats, bar height indicates the mean over rats, and error bars indicate standard error over rats. n.s. indicates not significant.

## Data Availability

All data will be available on the https://memlab.sitehost.iu.edu/ but we are also happy to provide access to any data or code covered in this manuscript to those who contact us directly.
